# Patient Perspectives on Communication Pathways After Orthopedic Surgery and Discharge and Evaluation of Team-Based Digital Communication: Qualitative Exploratory Study

**DOI:** 10.2196/49696

**Published:** 2024-03-29

**Authors:** Lili Worre Høpfner Jensen, Ole Rahbek, Rikke Emilie Kildahl Lauritsen, Søren Kold, Birthe Dinesen

**Affiliations:** 1 Interdisciplinary Orthopaedics Orthopaedic Surgery Department Aalborg University Hospital Aalborg Denmark; 2 Laboratory for Welfare Technologies – Digital Health & Rehabilitation Sport Sciences – Performance & Technology, Department of Health Science and Technology Aalborg University Aalborg East Denmark

**Keywords:** digital communication, patient-provider communication, continuity of care, interdisciplinary communication, hospital discharge, orthopedic surgery, postoperative care, text messaging, mobile phone

## Abstract

**Background:**

The transition from hospital to home after orthopedic surgery requires smooth communication and coordination between patients and their team of care to avoid fragmented care pathways. Digital communication is increasingly being used to facilitate easy and accessible asynchronous communication between patients and health care professionals across settings. A team-based approach to digital communication may provide optimized quality of care in the postoperative period following orthopedic surgery and hospital discharge.

**Objective:**

This study was divided into two phases that aimed to (1) explore the perspectives of patients undergoing orthopedic surgery on current communication pathways at a tertiary hospital in Denmark and (2) test and explore patients’ experiences and use of team-based digital communication following hospital discharge (eDialogue).

**Methods:**

A triangulation of qualitative data collection techniques was applied: document analysis, participant observations (n=16 hours), semistructured interviews with patients before (n=31) and after (n=24) their access to eDialogue, and exploration of use data.

**Results:**

Findings show that patients experience difficult communication pathways after hospital discharge and a lack of information due to inadequate coordination of care. eDialogue was used by 84% (26/31) of the patients, and they suggested that it provided a sense of security, coherence, and proximity in the aftercare rearranging communication pathways for the better. Specific drivers and barriers to use were identified, and these call for further exploration of eDialogue.

**Conclusions:**

In conclusion, patients evaluated eDialogue positively and suggested that it could support them after returning home following orthopedic surgery.

## Introduction

Across the health care system, digital communication is being implemented as an addition to traditional communication pathways [1,2]. Digital communication is a form of eHealth [3] that facilitates asynchronous 2-way text messaging between patients and health care professionals (HCPs). Digital communication is typically facilitated through email [4,5]; secure text messaging in patient portals [2,6]; or as a feature in mobile health apps developed for specific purposes, for example, postoperative monitoring [7,8] and neonatal tele-homecare [9,10]. Establishing the effects of using digital communication is still challenging [11,12]; however, an increasing number of studies suggest that it can support patients in taking care of their own health [12] and address unmet communication needs after hospital discharge [13,14]. When digital communication is used with the purpose of facilitating team-based communication across settings, studies indicate that it may contribute to improving continuity of care (COC) in transitions from hospital to home [14-16]. COC is essential for patients undergoing complex and long-term procedures [17]. Patients who receive care across time and settings are susceptible to fragmented care, and the absence of consistent professional support and communication may lead to neglect that ultimately affects patient safety [18-21]. Because of the growing population in need of orthopedic surgery, workforce shortage [22], and optimized surgery techniques, patients undergoing orthopedic surgery are discharged earlier [23]. Day surgery is increasingly used, and even patients undergoing complex treatments are hospitalized for a shorter time. Common to patients undergoing orthopedic surgery is a need for continuing rehabilitation across settings, supported by adequate communication and home symptom monitoring between follow-up visits [24,25]. Even so, only a few studies have addressed the use of team-based digital communication involving patients and HCPs across settings, and primarily in other patient populations, such as patients with cancer [14,15,26] and children with cerebral palsy [27]. To our knowledge, no studies have investigated the use of team-based digital communication after hospital discharge in orthopedic surgery, although these patients often have long periods of rehabilitation, where cross-disciplinary and cross-sectoral communication is pivotal [28].

Therefore, the aim of this study was to explore the perspectives of patients undergoing orthopedic surgery on current communication pathways (phase 1) and to subsequently test and explore their experiences and use of a team-based digital communication solution (eDialogue) to evaluate whether the solution can support their needs after hospital discharge (phase 2).

## Methods

### The eDialogue Intervention

**Figure 1:**
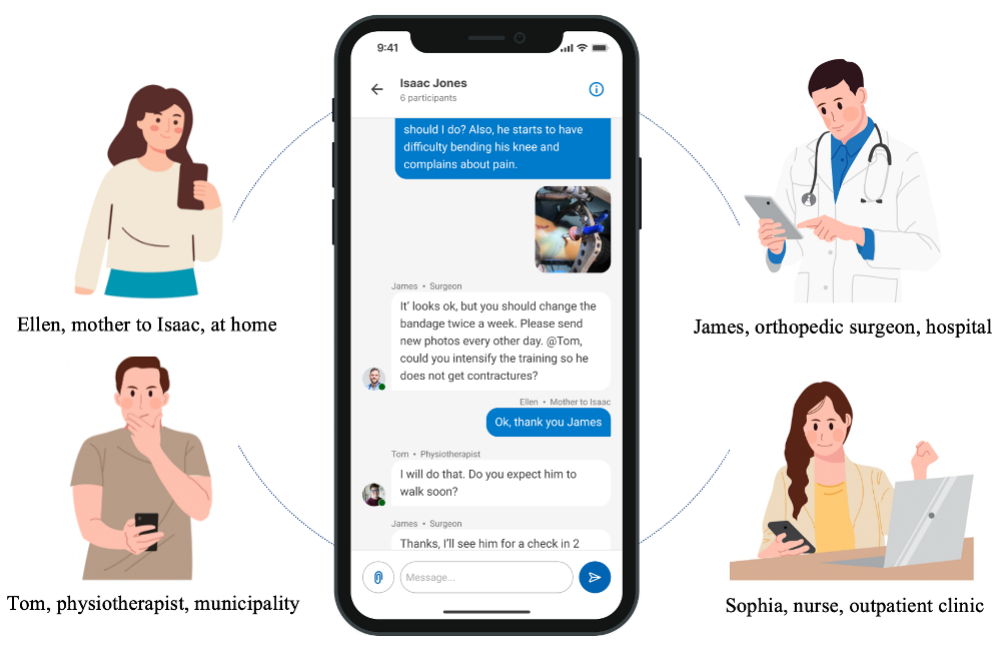
Illustration of the team-based digital communication (eDialogue) used in this study, where patients and health care professionals across settings could text and send photos to communicate about postdischarge issues.

The technical solution used in this study was a simple General Data Protection Regulation–compliant solution, developed for team-based communication, that lets users chat directly with each other with texts and photos (“LetDialog” by Visma) [29] (Figure 1).

The solution was accessed through an app for smartphones or through a website. Users could choose how they accessed it individually. To ensure compliance with the current legislation, user profiles were created with a digital signature (NemID), and the digital dialogues were stored in a secure cloud-based solution. A data processor agreement was made among the North Denmark Region, Aalborg University Hospital, and Visma before this study.

The features were basic asynchronous text messaging and exchange of photos. Photos could be taken directly or uploaded and sent through the solution for review by the health care team. Team-based digital communication was organized in teams, defined by the individual patient, in a shared chat. Notifications were sent to all the participants when there were new posts. Key HCPs from the orthopedic surgery department at the hospital were identified and recruited for participation before the study (surgeons, nurses, and physiotherapists). Other HCPs from municipal or private settings were recruited ad hoc and based on patients’ wishes (eg, physiotherapists from the municipality).

### Study Design

The study was exploratory, using a triangulation of qualitative data collection techniques, including document analysis, participant observations [30], semistructured interviews [31], and use data, with the purpose of obtaining in-depth knowledge of patients’ perspectives and the context.

### Theoretical Framework

The theoretical framework for this study was inspired by the concepts of COC [17,32], which is used as a measure of quality of care in health care transitions. COC includes *informational continuity*, described as the use of medical or personal information to provide appropriate care over time; *management continuity*, which refers to the provision of timely, coordinated, and complementary services that are responsive to patients’ needs to connect care over time; and *relational continuity*, which involves the consistency and quality of relationships between patients and providers as a means of connecting care over time [32]. All 3 dimensions should be integrated to achieve COC, and thus, COC is maximized when planning for patient-provider continuity, information exchange, and seamless coordination of services in the period of transition from hospital to home [32-34]. For this study, COC has inspired the data collection and analysis of interviews and observations as well as the use of team-based digital communication to prevent fragmented care experiences after hospital discharge.

### Participants and Setting

The study was conducted at the Orthopedic Surgery Department of Aalborg University Hospital, Denmark. The recruitment of participants began in May 2021 and ended in November 2021. The final follow-up interviews were conducted 2 months later in January 2022.

In phase 1, participants were recruited consecutively based on predefined inclusion criteria: (1) patients, or their parents if the patient was aged <15 years, undergoing deformity correction (DC) surgery or anterior cruciate ligament (ACL) reconstruction; (2) those who were able to read and write Danish; (3) those who were discharged to their own home and had planned follow-up in the outpatient clinic; and (4) those who owned a smartphone and had access to a secure digital signature. The exclusion criteria were (1) those who were not able to understand Danish and (2) those who were not cognitively able to participate in interviews.

The 2 patient groups, DC and ACL, were selected because they represent 2 different orthopedic surgical care pathways. Involving both patient groups allowed us to gain an insight into the different needs of patients undergoing orthopedic surgery. ACL is performed as a day surgery (ie, discharge on the same day), whereas patients in the DC group most often have longer hospitalizations and prolonged treatments.

The same recruitment procedure was used for patients undergoing DC or ACL. The patients were approached by secretaries at the hospital with an invitation to participate. If the patients agreed to be called by phone with information about participation in the study, the first author (LWHJ) would call them to provide oral participant information. Written participant information was then sent by email, and the patients were given time to consider participation. One patient did not want to participate after receiving oral information due to a lack of mental capacity to participate in the interviews. Another patient could not be contacted by telephone after he had initially registered his telephone number. Both patients were from the ACL group.

In phase 2, patients and parents (if the patient was a minor) were onboarded to eDialogue on the day of discharge. The orthopedic surgeon, who had performed the surgery, was invited to join the patients’ dialogue, as were nurses from the outpatient clinic and physiotherapists across sectors who were involved in the patient’s care and rehabilitation after discharge. Thus, the patients were connected with known HCPs and were able to use eDialogue as needed from the day of discharge until 2 months after discharge. The patients could send texts and photos whenever it suited them, but they were told that a 24-hour response time on weekdays (Monday to Friday) would be aimed for. As such, messages sent during weekends and holidays would be responded to on the next weekday. It was pointed out, both verbally and in the participant information letter, that in case of emergency, patients should not use the solution but instead call, as they usually would have done before access to eDialogue. Thus, eDialogue was an addition to traditional communication channels (eg, telephone calls and email) and an extra opportunity for communication after discharge.

### Data Collection

A triangulation of data collection techniques was performed to achieve exhaustive knowledge of current communication pathways, patients’ perspectives, and their experiences with eDialogue.

#### Phase 1

First, document analysis was performed on documents and guidelines for postdischarge communication between patients and HCPs followed by participant observations of workflows (n=16 hours). The aim of the document analysis was to obtain knowledge of the policies and context of the study. The aim of observations was to document the current communication pathways for patients following hospital discharge. Participant observations were performed by LWHJ and followed a predefined observation guide [30]. Observations were carried out at the orthopedic surgery ward and the outpatient clinic at the hospital and documented in Word files (Microsoft Corp). This involved, for example, secretaries’ handling of incoming phone calls from patients, registration of patient inquiries, procedures for passing on messages to nurses and orthopedic surgeons, and HCPs’ calls with patients. In addition, existing systems for communication with discharged patients were reviewed, including written communication to patients via “E-box,” (a secure digital mail system for communication from Danish authorities) correspondence between HCPs across hospitals and municipalities in the local electronic health record, and interprofessional communication related to patients’ phone calls.

Second, semistructured interviews were conducted at the point of inclusion for each participant (N=31). The aim was to explore patients’ and parents’ perspectives on current communication pathways. Interviews were performed using video 5 to 7 days before surgery for patients from the ACL group (n=14) and physically at the ward for patients and parents from the DC group (n=17) because they were all hospitalized in connection with their operation. All interviews were conducted by LWHJ based on a predefined semistructured interview guide (Multimedia Appendix 1). The guide was developed based on the theoretical framework for this study and combined with exploratory questions. It was pilot-tested in 2 patients similar to the study participants and revised accordingly. The interviews were carried out until data saturation had been reached, defined by the point where no new insights into participants’ responses occurred, indicating the achievement of a comprehensive understanding of the participants’ perspectives [31]. The interviews were audio recorded using a digital voice recorder (DM-450; Olympus) and lasted for 40 to 60 minutes. They were continuously transcribed and documented in Word files. During and at the end of each interview, key points were summarized to ensure the credibility of the meanings expressed.

#### Phase 2

Semistructured follow-up interviews were performed with the same patients and parents 2 months after hospital discharge (24/31, 77%). The aim was to explore their experiences of using eDialogue for team-based communication in the postdischarge period. The interviews were performed by LWHJ, audio recorded, and followed a predefined interview guide that was pilot-tested (Multimedia Appendix 1). The interviews were conducted until data saturation was reached for each patient group [31]. They lasted between 30 and 60 minutes. Both users and those who did not use eDialogue after getting access were interviewed. A total of 6 patients (DC: n=3; ACL: n=3) were reached by phone, their experiences were discussed, and a short report was written. Nothing new emerged from these conversations. One parent of a child from the DC group was lost to follow-up as she did not return our calls. Interviews were performed face-to-face at the ward or digitally based on the preferences of the participants. Participants were most likely to choose web-based interviews due to convenience and distance to the hospital, and data collection was conducted at the same time as the COVID-19 pandemic.

Use data of eDialogue was collected through registration of events and manual counts of messages exchanged in all digital dialogues. Data included the total number of messages exchanged in eDialogue during the 2-month study period, the number of text messages and photos sent by patients or parents, and the number of text messages that actually needed a reply from HCPs. In addition, the distribution of text messages per week per patient group was collected and displayed to show the differences between groups. Content analysis [31] of the messages sent by the patients and the parents was performed to provide insight into question categories as well as how they were distributed between the patient groups.

### Data Analysis

Data analysis was carried out in NVivo (version 20.6.2; Lumivero), inspired by Brinkmann and Kvale [31], with the aim of achieving an in-depth understanding and connection of the participants’ expressed perspectives on current communication pathways (phase 1) and experiences using eDialogue (phase 2).

Separate data analyses were carried out for phase 1 and phase 2 and for each patient group (DC and ACL), all involving 3 steps: meaning coding, meaning condensation, and meaning interpretation (Textbox 1).

In phase 1, observational data were integrated into the data set to enhance the understanding of existing communication pathways for patients in need of postdischarge contact.

Use data from eDialogue were analyzed and presented using simple descriptive statistics and basic content analysis to present the overall question categories.

The reporting of this study followed the Consolidated Criteria for Reporting Qualitative Research checklist [35].

**The process of the thematic analysis.**

**Meaning coding**

- Coding of the transcribed interviews was initially performed individually by 2 of the authors (LWHJ and REKL) by randomly selecting 4 interviews from each patient group and from each phase. Coding was conducted with an inductive approach with the aim of reflecting the meanings expressed by the participants.
- To achieve intersubjectivity before and during the analysis, interviews were individually read and reread by LWHJ and REKL, notes were made for initial ideas for codes, and these were then compared and discussed until agreement. In phase 1, this resulted in 21 codes in the deformity correction (DC) group and 18 codes in the anterior cruciate ligament (ACL) group. In phase 2, we identified 18 codes in the DC group and 15 codes in the ACL group. LWHJ continued the data analysis of the remaining interviews by applying the same codes to the entire data.

**Meaning condensation**

- The codes were then reread and condensed in discussion with REKL and the last author (BD) through several iterations, and this process resulted in 12 codes for the DC group and 8 codes for the ACL group in phase 1 and 11 codes for the DC group and 7 codes for the ACL group in phase 2. We merged and we left behind codes that did not directly address the research questions or were only described vaguely by 1 participant.
- The remaining codes were then discussed with all authors to achieve further condensation and to define and name subthemes and themes that would capture the essence of the data. It was clear to the authors that the 2 patient groups had expressed similar perspectives on the phenomena of interest, and therefore, codes could be merged between the 2 groups in this step. Theme generation was based on a systematic identification and organization of recurring patterns, topics, or concepts within the data set. This process resulted in 3 overall themes and 6 subthemes for phase 1 and 3 overall themes and 3 subthemes for phase 2.

**Meaning interpretation**

- Themes were defined and described narratively, and data extracts were chosen for presentation in the manuscript before writing the findings.

### Ethical Considerations

Before the study started, the Ethics Committee of Northern Jutland was approached, and it was found that the study did not require approval, as eDialogue was an extra opportunity for patients to communicate directly with their team of HCPs across sectors. This was confirmed by email on March 18, 2021 (2021-000438). The study was registered with the Regional Committee on Health Research and approved (ID number 2021-057). All participants received thorough oral and written information and guidance in the use of eDialogue before discharge. The study followed the Helsinki Declaration, and the participants signed an informed consent form and were able to leave the study without explanation or effects on usual care. All patients or parents had access to eDialogue for 2 months after hospital discharge. If they wanted, patients were allowed to keep the possibility of eDialogue with their team of HCPs after 2 months and until their follow-up in the outpatient clinic was completed. An administrator from the project group was passively present in all dialogues to continuously observe whether the patients used the solution for emergencies against the given advice.

## Results

### Participants’ Characteristics

Table 1 provides the baseline description of the 31 patients included in this study. The patients were recruited from 2 different subgroups of orthopedic surgery: DC (17/31, 55%) and ACL (14/31, 45%).

The patients in the DC group were, for example, patients with malalignment or limb length discrepancy, and they were all hospitalized for >1 day. The patients in the ACL group were all treated with ACL reconstruction, and they had the procedure performed as day surgery. Of the 14 patients with ACL injuries, 7 (50%) had a concurrent meniscal injury.

Across the groups, most patients were male (22/31, 71%), and patients ranged in age from 1 to 59 years. Patients from the DC group were discharged from the hospital after an average of 6.1 (range 1-9) days, and patients from the ACL group were all discharged on the same day of surgery (<9 hours of admission). All the included patients were discharged to their own home. Of the 31 patients included, 14 (45%) had previously undergone orthopedic surgery at Aalborg University Hospital, and thus, they were able to reflect on previous experiences with postdischarge communication during the initial interviews in phase 1. In the DC group, 5 patients lived outside the North Jutland Region.

A total of 42% (13/31) of the patients were children aged <15 years, and thus, their parents were the primary users of eDialogue. Therefore, the baseline characteristics of all users of eDialogue are presented in Table 2.

The table shows 33 users in total because 2 patients aged 16 and 17 years had a parent joining the dialogue with them. Of the 13 parents who were users of eDialogue with or on behalf of their child, 77% (10/13) were female (mothers). The mean age of the parents was 43 (range 37-48) years on the day of discharge. All users of eDialogue used a smartphone on a daily basis.

**Table 1:** Characteristics of all patients across groups (DC^a^, n=17; ACL^b^, n=14; N=31).

Characteristics		Values
**Sex (DC/ACL), n (%)**		
	Female	5 (29)/4 (29)
	Male	12 (71)/10 (71)
**Age at discharge (years), mean** **(range)**		
	DC	19.2 (1-59)
	ACL	29.1 (17-46)
**Length of hospital stay, mean (range)**		
	DC	6.1 (1-9) days
	ACL	1 (7-9) hours
**Previously had orthopedic surgery (yes/no), n (%)**		
	DC	12 (71)/5 (29)
	ACL	2 (14)/12 (86)
**Highest education level (DC/ACL), n (%)**		
	Primary or high school	12 (71)/5 (36)
	Vocational education (skilled worker)	2 (12)/2 (14)
	Short education, 2-3 years	1 (6)/2 (14)
	Bachelor’s degree, 3-5 years	2 (12)/4 (29)
	Academic education, 5-8 years	0 (0)/1 (7)
**Work status (DC/ACL), n (%)**		
	Student	13 (76)/7 (50)
	Unemployed	1 (6)/2 (14)
	Employed	3 (18)/5 (36)
**Civil status (DC/ACL), n (%)**		
	Living alone	3 (18)/4 (29)
	Cohabiting	14 (82)/10 (71)

^a^DC: deformity correction.

^b^ACL: anterior cruciate ligament.

**Table 2:** Baseline characteristics of all users of eDialogue (DC^a^, n=18; ACL^b^, n=15; patients and parents; N=33).

Characteristics		Values
**Distribution of users (DC/ACL), n (%)**		
	Patients	6 (33)/14 (93)
	Parents	12 (67)/1 (7)
**Sex (DC/ACL), n (%)**		
	Female	12 (67)/5 (33)
	Male	6 (33)/10 (67)
**Age at discharge (years), mean (range)**		
	DC	39.8 (16-59)
	ACL	28.8 (17-46)
**Highest education level (DC/ACL), n (%)**		
	Primary or high school	2 (11)/5 (33)
	Vocational education (skilled worker)	3 (17)/2 (13)
	Short education, 2-3 years	3 (17)/2 (13)
	Bachelor’s degree, 3-5 years	8 (44)/5 (33)
	Master’s degree, 5-8 years	2(11)/1 (7)
**Work status (DC/ACL), n (%)**		
	Student	2 (11)/7 (47)
	Unemployed	1 (6)/2 (13)
	Employed	14 (78)/6 (40)
	Disability pensioner	1 (6)/0 (0)
**Civil status (DC/ACL), n (%)**		
	Living alone	4 (22)/3 (20)
	Cohabiting	14 (78)/12 (80)

^a^DC: deformity correction.

^b^ACL: anterior cruciate ligament.

### Phase 1: Perspectives on Current Communication Pathways

#### Themes and Subthemes

Through the initial interviews, 3 themes and associated subthemes were revealed across the groups. Overall, patients and parents from the DC and ACL groups had similar experiences of, and perspectives on, current communication pathways. However, some subthemes were more prominent in one group than the other. This is illustrated by showing how many patients and parents from each group expressed experiences related to the specific subtheme (Table 3).

**Table 3:** Themes and subthemes of patients’ and parents’ perspectives on current communication pathways with HCPs^a^ after hospital discharge (N=31).

Themes and subthemes		DC^b^ (n=17), n (%)	ACL^c^ (n=14), n (%)
**Difficult** **communication pathways**			
	Doubts about who to contact and when	8 (47)	7 (50)
	Withhold questions or forget to ask	7 (41)	9 (64)
**Lack of information due to inadequate coordination of care**			
	Knowledge is not shared sufficiently	8 (47)	6 (43)
	Hard to be “the messenger” between HCPs	9 (53)	5 (36)
**Relations and communication provide “peace of mind”**			
	Relational continuity matters	15 (88)	4 (29)
	Contacts provides a sense of being cared for	10 (59)	2 (14)

^a^HCP: health care professional.

^b^DC: deformity correction.

^c^ACL: anterior cruciate ligament.

#### Difficult Communication Pathways

Most patients and parents expressed frustrations related to difficult communication pathways when they needed contact with HCPs. They were in doubt about who to contact regarding specific issues both before and after surgery and discharge:

It was like a week after discharge, and I didn’t know who to ask. Should I contact the department, the outpatient clinic or my own physician? I didn’t know that. They kept telling me to call a new location.

Mother of patient 2, DC

The patients also described how they would often forget to ask questions at the outpatient clinic or they would withhold questions because they found it difficult to assess whether their issues were “severe enough” to take up HCPs time. A patient explains how it had previously led to concerns and worsening of symptoms:

I couldn’t lift up my leg like I had been able to before...The next morning, the knee was barely visible due to swelling. Well, I should probably have done something the day before, but I didn’t. You just know that when you call the hospital, you must go through several people, and I don’t want to be a nuisance either.

Patient 4, DC

#### Lack of Information Due to Inadequate Coordination of Care

Patients in the ACL group highlighted a lack of information before surgery. Similarly, they described missing information in the first weeks after discharge, before their postoperative follow-up visit, and before starting rehabilitation with a physiotherapist:

Actually, I didn’t know what I was supposed to do. Maybe I didn’t ask enough questions before discharge. The first week (after discharge) I didn’t do anything. I was wearing this DonJoy bandage and I didn’t put stress on my leg or anything. And it turns out that I really should have done that.

Patient 1, ACL

They had questions about rehabilitation and restrictions associated with the operation, and this led to Google searches, which usually left them more confused:

I felt like I was in a no man’s land and didn’t really know what to do.

Patient 3, ACL

In the DC group, the patients and the parents described how knowledge is not shared across sectors in a sufficient and timely fashion. The fact that HCPs in the municipality did not have specialty-specific knowledge, as did those from the hospital, was perceived as unsafe and uncertain. They described situations in which home care nurses or physiotherapists had little or no experience with their treatment and care. That placed a massive burden on the patients or the parents to be in “control” of everything. Lack of information and coordination across sectors also led to confusion regarding the rehabilitation, for example, when the physiotherapist understood the rehabilitation plan differently than the patient remembered it. The patients and the parents from the DC group pointed out how they become the “messengers” and thus responsible for passing on information between the hospital and municipal providers. They viewed this as burdensome, expressing insecurity about accurately conveying all crucial information:

It’s the fact that it is our interpretation of what is heard. You know, it is not necessarily medical language that we pass on to the next professional.

Mother of patient 13, DC

The physiotherapists often ask questions like “what did the surgeon say?” But when you have no professional knowledge, and you are busier with being there for your child, then there might be things I do not remember or consider as being important.

Mother of patient 12, DC

#### Relations and Communication Provide “Peace of Mind”

Patients and parents from both groups highlighted the importance of the relationship and communication with HCPs. However, they had different perceptions of their actual needs. For the patients in the ACL group, the most important thing was that the HCPs were “competent.” This was also valid in the DC group, but they unanimously expressed that the relationship and contact with known HCPs were just as important to them. The mother of a boy, who had been through several operations throughout his childhood, described what the relationship between her son and the HCPs at the hospital meant:

It gives, well, it gives you peace. It gives peace of mind even before you have to leave home (to attend surgery or follow-up visit). He can say: “Well, now we’re going home to Aalborg again soon,” and people will say “You don’t live in Aalborg, do you?.” And then he would respond: “Well, a lot of my time, I do.”

Mother of patient 7, DC

The same perspective was elaborated by the mother of another boy:

I think it’s about safety, trust, and recognizability, and we don’t refer to it as the “doctor,” we say we’re going to see him (the surgeon) or her (the nurse).

Mother of patient 15, DC

During the initial interviews, it became clear that some patients undergoing long-term treatments in the DC group already used email or SMS text messaging for communication with the orthopedic surgeon or the physiotherapist. This was described as a workaround because traditional communication pathways did not meet their needs, such as calling the secretary, who would leave a note for the nurse or the surgeon to call the patient. The patients and the parents expressed that it made them feel supported, and thus, they largely understood the intention of eDialogue. When asked about their expectations of eDialogue, most patients and parents who had previous experiences with orthopedic surgery expressed that they wished they had had the opportunity of team-based digital communication the first time. Thus, they expected that their previous experiences of “being a patient” would minimize their need for eDialogue at this time.

### Phase 2: Experiences With, and Use of, eDialogue After Discharge

#### Themes and Subthemes

All 31 patients or their parents included in this study were given access to eDialogue for 2 months after discharge with their team of HCPs across sectors. Interviews with 77% (24/31) of the patients and parents led to 3 overall themes and associated subthemes identified across the groups. As in the initial interviews, some subthemes were more prominent in one group than the other and thus highlighted in the table (Table 4).

**Table 4:** Themes and subthemes of patients and parents’ experiences of using eDialogue with HCPs^a^ after discharge (n=24).

Themes and subthemes		DC^b^ (n=13), n (%)	ACL^c^ (n=11), n (%)
**Digitally enhanced coherence and proximity**			
	A sense of security at home	13 (100)	7 (64)
	Sharing knowledge between patients and HCPs	9 (69)	5 (45)
**Drivers and barriers to use**			
	Recognizable, informal tool and easy to use	11 (85)	8 (73)
	To “be invited” to dialogue by HCPs allows use	6 (46)	4 (36)
	Worry about overburdening HCPs	10 (77)	2 (18)
**eDialogue rearranges communication pathways**			
	Reduces the need for phone calls	12 (92)	6 (55)
	Text messages and photos are adequate	9 (69)	7 (64)

^a^HCP: health care professional.

^b^DC: deformity correction.

^c^ACL: anterior cruciate ligament.

#### Digitally Enhanced Coherence and Proximity

Across groups, patients and parents unanimously reported that the possibility of easy and direct communication with HCPs after discharge provided them with a sense of security at home. Although eDialogue was used sparingly by some patients, the possibility made them feel at ease during the rehabilitation period. For the patients who used eDialogue more, it was expressed that it helped them get through the first period after discharge because they felt “closer” to the HCPs and as if they had a constant “back up”:

For me, it is very much about security, I almost feel that I have the surgeon by my side all the time. The first time (of surgery and discharge), I felt that he was far away.

Patient 4, DC

The patients in the ACL group appreciated the opportunity to ask questions, but the need for communication was most evident in the first weeks after discharge and before the first clinical follow-up and exercise sessions with physiotherapists:

Before my first checkup, I encountered some problems that I really wanted answered, so that I didn’t have to go and wait and worry if there was something wrong. It was solved immediately in eDialogue.

Patient 8, ACL

For the patients and the parents in the DC group, eDialogue specifically helped HCPs share important information across sectors. They described how no longer being responsible for passing on information between the surgeon and the physiotherapist at the municipality brought relief and was highly appreciated:

Then we could see that they had the dialogue and then we knew that when we showed up for training next time, the physiotherapist knew it, so we didn’t have to explain, which we found difficult anyway.

Father of patient 10, DC

In other cases, the patients described how municipal HCPs would use eDialogue indirectly to keep updated with the patient’s progress just by reading the messages exchanged between the HCPs from the hospital and the patient. This provided a basis for a common point of view at the patient’s next training session.

The parents of minor children described how they used eDialogue to calm their child or explain the treatment plan to them by reading them messages from HCPs.

#### Drivers and Barriers to Use

In both groups, the patients and the parents agreed that eDialogue presented as a recognizable and informal tool that was easy to use and that this promoted their use. The short response time was also highlighted as a main reason to use eDialogue:

I don’t remember a day has passed, more like minutes or hours. So, it’s been cool. It would never have been the case if I had to call.

Patient 1, ACL

Few patients experienced a late or no response. If it happened with their first question, they explained that it made them lose courage to use eDialogue another time. In general, the patients and the parents felt that the use of eDialogue was less intrusive than calling, but they also expressed worry about overburdening the HCPs. By contrast, they expected HCPs to manage their working hours themselves and assess when they had the time to respond:

To begin with, I thought that I would not burden the system unnecessarily...but it probably became a little more urgent and I worried about the way he was feeling, so I texted them and got a reply shortly after.

Mother of patient 12, DC

No patients expected answers out of hours, but some sent messages at these times to be relieved. However, they all emphasized that they could have waited for a response until the next weekday. A patient from the ACL group described her reflections about sending a message on a Friday night:

And of course, I thought, Oh no, now I hope he doesn’t feel obliged to answer, but I also thought that they must be professional and decide for themselves.

Patient 11, ACL

Some patients and parents described how, before discharge, some HCPs would urge them to use eDialogue if needed and that the feeling of being invited made them more inclined to use it after coming home. The patients from the ACL group also described how eDialogue opened up the possibility to ask about “minor issues,” which they might not have called about.

Among nonusers or those who used eDialogue sparingly, it was expressed that they simply did not have the need, as everything went as planned. Nonuse was also attributed to having frequent follow-ups at the outpatient clinic or attending physiotherapy several times a week.

#### eDialogue Rearranges Communication Pathways

The patients and the parents highlighted how the use of eDialogue had prevented phone calls or additional physical attendance after discharge; this was particularly prominent for the patients in the DC group:

Well, to start with we used eDialogue quite a bit I would say. As soon as we had any questions, we texted them and did not need any other forms of communication.

Mother of patient 8, DC

In a few cases, messages in eDialogue developed into a need for phone calls or an extra checkup in the outpatient clinic. The time of the phone call or attendance was then arranged through eDialogue. However, digital communication was perceived as adequate in most cases. There were instances where follow-up questions from HCPs were necessary, yet patients quickly felt understood and equally comprehended the answers they received:

Although we have not spoken on the phone, I have received sufficient information and I also feel that I have managed to communicate well.

Mother of patient 1, DC

A patient from the ACL group described how eDialogue was used as an extra contact for a him to “fully guard” himself. He was in doubt if the photo sent in eDialogue could show his concerns regarding the surgical site clearly enough, and therefore, he contacted his general practitioner and texted the team in eDialogue at the same time:

There was a situation where I had sent a message in the morning, and so, I thought I might as well, while there was still phone time at the GP, call to see if he had an available appointment. Then I came to my GP, and actually got exactly the same answer as I received on the phone (eDialogue) an hour later. So, it wasn’t something that was needed as such, but now that I had the opportunity, I thought I might as well do it.

Patient 8, ACL

No patients expressed feelings of being misunderstood in their communication with HCPs in eDialogue. They experienced digital communication as being sufficient for their needs; however, they reflected on the risk of misunderstandings when communicating via texts:

I think it’s a much more optimized way of doing it, because I don’t need a physical conversation by phone. I’m fine with texting, but obviously there can be some misunderstandings or something that can go wrong and then you have to call.

Mother of patient 15, DC

The use of photos was mentioned as being very important to support texts. A few patients explained that they lacked the possibility of sending and receiving videos; however, they emphasized that it was not a necessity for their use:

If I hadn’t been able to send photos, then maybe I would have had to explain something visual by phone, and then I would have had to come in for a checkup, and then I would have wasted a whole day.

Patient 1, ACL

Video could be nice, but then again, the photos could effectively illustrate how the position of her leg is and show how much she has actually been able to stretch, in what positions it hurts, and so on.

Mother of patient 17, DC

The mother of a minor patient explained how she used eDialogue as a photo diary to keep the HCPs across sectors updated on the progress of her son’s surgical wound:

So, when she (the home care nurse) came and changed the dressings, we took some photos before she put on new ones, and then we kind of had it (photos) from time to time and could follow how it progressed...It was smart as hell, and when it wasn’t the same home care nurse coming by, we showed them the photos and at the same time kept the surgeon at the hospital up to date.

Mother of patient 15, DC

#### Use of eDialogue 2 Months After Discharge

The need for support and communication for both patient groups after discharge was expressed through the actual use of eDialogue (Table 5).

**Table 5:** Patients’ and parents’ use of eDialogue 2 months after hospital discharge.

		Total number of messages, n	Average number of messages per patient, n	Maximum number of messages per patient, n
**DC^a^ (n=17)**				
	All text messages exchanged^b^	338	19.9	54
	Text messages sent by patients	189	11.2	34
	Actual questions that needed a reply^c^	128	7.5	20
	Photos sent by patients^d^	127	7.5	53
**ACL^e^ (n=14)**				
	All text messages exchanged	126	9.0	36
	Text messages sent by patients	68	4.9	19
	Actual questions that needed a reply^f^	55	3.9	14
	Photos sent by patients^c^	13	0.9	6

^a^DC: deformity correction.

^b^The total number of text messages exchanged between patients and health care professionals (HCPs) 2 months after discharge.

^c^Text messages sent from the patient or their parents to the HCPs in eDialogue. The minimum number of messages or photos sent per patient was 0, as some patients did not use eDialogue at all.

^d^Actual questions that needed a reply from the HCPs are the number of individual text messages from patients or parents that were formulated as a question; thus, this does not include the back-and-forth 2-way communication that 1 question could lead to (eg, saying thank you).

^e^ACL: anterior cruciate ligament.

^f^Photos refer to the number of photos taken by the patients or parents and sent for review by the HCPs.

Of the patients or their parents, 88% (15/17) in the DC group and 79% (11/14) in the ACL group used eDialogue to ask questions to HCPs after discharge. In the DC group, 13 (87%) of the 15 active users used photos, and in the ACL group, 5 (45%) of the 11 active users sent photos to support communication. Upon inclusion in the study, the patients and the parents were informed that they could expect a response time of 24 hours during the weekdays. This was complied with in 96.2% (176/183) of the cases where a message that required a response from HCPs was sent, and the distribution was equal across groups.

Among users of eDialogue in the DC group, the minimum number of per-patient questions that needed a reply from HCPs was 2, and the maximum was 20. For the ACL group, there was a minimum of 1 and a maximum of 14 questions that needed a reply in 1 dialogue. Thus, there was a marked difference in the individual’s use of eDialogue during the study period in both groups.

Most of the communication took place from Monday to Friday; thus, 84.7% (155/183) of the questions that needed a reply from the HCPs were sent and replied to during the weekdays.

**Figure 2:**
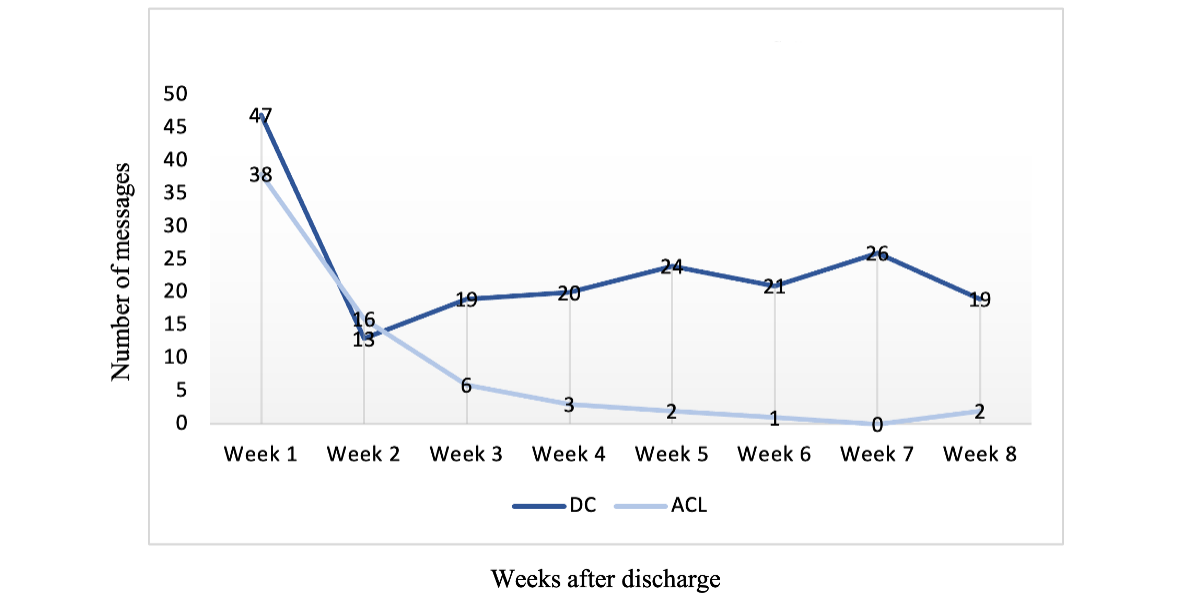
The number of individual text messages sent from patients or parents to the health care professionals in eDialogue per week 8 weeks after discharge. ACL: anterior cruciate ligament; DC: deformity correction.

**Figure 3:**
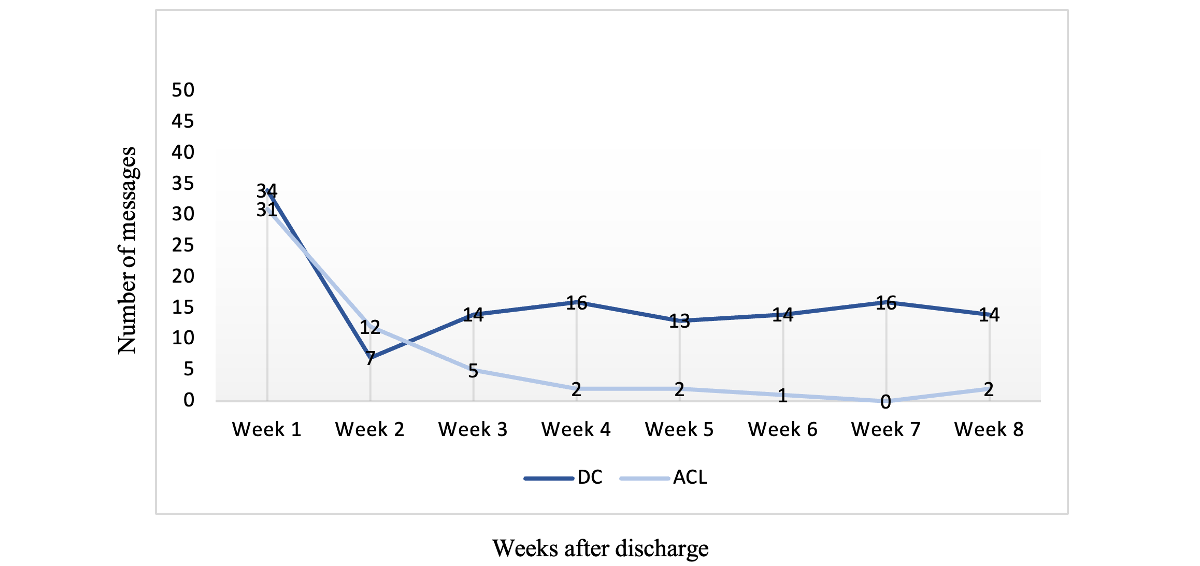
The number of messages sent by patients or parents that required a response from health care professionals, that is, messages phrased as a question, per week 8 weeks after discharge. ACL: anterior cruciate ligament; DC: deformity correction.

The patients and the parents in the DC group used eDialogue throughout the 2 months (Figures 2 and 3), and 15 (88%) of the 17 patients requested to keep on using it after the data collection stopped at 2 months. The patients in the ACL group primarily used eDialogue for the first 2 to 3 weeks after discharge (Figures 2 and 3), and use then faded. Only 2 (6%) of the 31 patients or parents expressed a need to continue with eDialogue after 2 months.

Content analysis of the messages in eDialogue revealed 9 overall categories, including treatment-related issues, rehabilitation and restrictions, concerns about symptoms and complications, medication, psychological support, interdisciplinary and cross-sectoral dialogue, coordination and practical needs, updates and gratitude, HCP ask for feedback. The categories were identified across groups; however, some categories were more prominent in one group than the other (Multimedia Appendix 2).

## Discussion

### Principal Findings

#### Overview

This study explored the perspectives of patients undergoing orthopedic surgery on current communication pathways (phase 1), and their subsequent experiences of using eDialogue after discharge, as well as the actual use of the solution (phase 2).

In phase 1, we identified unmet needs among patients regarding communication with HCPs after discharge. The themes involved perspectives of difficult communication pathways, lack of information due to inadequate coordination of care, and that relation and communication provide “peace of mind.” In phase 2, the participants were set up to use eDialogue for 2 months after surgery and discharge, providing them access to direct digital communication with their individual health care team across settings. Through follow-up interviews, they articulated the following themes: digitally enhanced coherence and proximity, drivers and barriers to use, and that eDialogue rearranges communication pathways. Use data of eDialogue supported the experiences expressed in the interviews and provided an overview of the actual use. These findings will be discussed with the theoretical framework of COC and previous research.

#### Signs of Improved COC With eDialogue

Through initial interviews, the patients and the parents expressed a need for more clear communication pathways after discharge. A patient expressed that it felt like being in a “no man’s land.” As such, they lacked communicative support at home as well as optimized sharing of knowledge between the HCPs involved in their treatment and care across settings, indicating that informational and management COC is under pressure [32]. Similar findings are described in other studies on patients’ experiences of the transition from hospital to home following surgery [24,28], and this emphasizes the need to address communicative challenges around hospital discharge.

The patients and the parents in complex and long-term orthopedic treatments (DC) experienced a greater need for continuous contact with their known health care team than those undergoing day surgery (ACL). Thus, the relationship, trust, and mutual understanding with the HCPs were described as being of great importance for their experience of security. For these patients, access to eDialogue was particularly useful, suggesting that eDialogue may play a role in facilitating relational COC. The patients in the ACL group, despite still having an unmet need for information, expressed that “less” would have been suitable for them. As digital communication becomes more prevalent in health care [1,2,4,5,7,9], comprehensive evaluations are crucial, including efficiency and optimal resource use considerations. Some patients may find less resource-intensive options, such as automated text message interventions, sufficient [36].

Through follow-up interviews, the patients and the parents across groups highlighted that eDialogue provided easy access to relevant HCPs and facilitated coherence and proximity after returning home, leading to “a sense of security.” These findings corroborate previous studies [14,37] and support our assumption that team-based digital communication may contribute to improving patients’ experiences of COC in transitions from hospital to home [32]. Other studies have also highlighted that COC is one of the factors that can be positively influenced by the use of team-based digital communication [15,16]. Voruganti et al [15] evaluated the feasibility of integrating a web-based communication tool for collaborative care in a pilot randomized controlled trial and found evidence indicating an increase in COC scores in the intervention group; however, the study was unpowered to show the effect statistically. Another study by Lindkvist et al [16] described how access to and use of an eHealth device for text-based communication, image exchange, and data reports between HCPs and parents of preterm infants or pediatric surgery was experienced positively in the transfer period from hospital to home. Moreover, they reported that parents felt it gave a sense of “shared responsibility,” which was also expressed by the patients and parents in this study. Thus, they highlighted that eDialogue facilitated the sharing of information, so they no longer had to be the ones passing on information and knowledge between HCPs. This was a role that they often disliked or mistrusted that they could fulfill adequately. The findings from this study indicate, in line with other studies [14-16], that digital team-based communication has the potential to set the framework for interdisciplinary and cross-sector collaboration that supports COC following hospital discharge. Whether team-based digital communication can actually enhance levels of COC to an extent where it can be measured remains to be investigated.

#### Patients Want to Communicate Digitally

As seen in other studies on digital asynchronous communication [15,16,38], use data demonstrated that most patients and parents across groups used eDialogue (26/31, 84%). The drivers to use eDialogue involved that the tool was recognizable and easy to use. Employing a messenger-like tool, made available to patients on their own smartphone, was a strength, as we did not encounter technical challenges as described in other studies, where devices were newly developed and delivered to participants [16]. The simple solution only allowed for communication in text and photos, and it may lack other options for patients who cannot use the text-based medium. Although previous studies involving text-based digital communication for health care purposes show that patients largely adopt this form of communication across settings and needs [4,10,37,39], digital inclusion in eHealth interventions is important to acknowledge both in regard to the hardware as well as patients’ ability to use the solutions [40]. As such, if the patients cannot use the tool, no value has been added. Other studies have integrated several means of communication into their solutions, including text, video, photos, and voice recordings, and found that video communication was especially useful [16,41,42]. This is in contrast to our findings, where patients expressed that the text-based medium was sufficient for them in the postoperative period. However, we acknowledge that eDialogue, as used in this study, may not be sufficient for all patients. When designing and implementing digital communication solutions, considering patients' literacy and eHealth literacy becomes crucial to ensure equal access to health care [40,43]. Integrating multiple communication modalities within a single solution could serve as a means to achieve this goal.

A driver mentioned in this study was the informality of the solution, and that it felt less interrupting than calling by phone. Similar results have been found in other studies of digital text-based communication [16,37], and this indicates a high degree of acceptance and usability of the solution from the patients’ perspectives. With an increasing level of smartphone use in the general population, digital communication becomes a more natural choice when patients need to contact providers. Thus, statistics show that the use of smartphones worldwide is increasing significantly, and in Denmark, it is estimated that 90% of all households own a smartphone [44]. As a barrier to use, the patients expressed concerns about wasting the HCPs’ time. This is important to consider when implementing solutions for digital communication. Our findings indicate that this may be offset by a more inviting approach from the HCPs, as some patients and parents expressed this as a facilitator to their use. Previous studies have pointed out the importance of clearly communicating response times when using digital communication [16,37]. Similar findings were highlighted in follow-up interviews of this study, where patients and parents described quick response times, or alternatively late responses, as a driver and a barrier, respectively.

Across groups, the patients and the parents expressed that eDialogue, despite only being an addition to existing communication channels, had rearranged the communication pathways significantly. This became obvious as the patients and parents described a reduced need to call the hospital, as they found eDialogue adequate and exhaustive for their needs. These findings corroborate previous studies showing a potential decrease in phone calls to the hospital after discharge when digital communication is being used [16,45]. By contrast, another study reported, in line with our study, that some questions asked by patients in a digital communication tool were not something they would have called about and thereby indicate that access to digital communication may contribute to an increased consumption of health care resources [16]. To evaluate the effect on resource use, a randomized controlled trial should be performed. Future studies designed to demonstrate the effects on health resource use are desired to shed light on whether digital communication actually reduces patients’ use of other forms of communication channels or adds on. In addition, it should be considered whether digital communication provides better quality, for example, defined as COC, patient satisfaction, and security for patients.

This study adds to the knowledge of patients’ perspectives on current communication pathways and the sparse evidence of their experiences and use of digital team-based communication, specifically in an orthopedic surgery setting. This may inform future interventions of team-based digital communication, from its application in clinical practice to organizational and management levels.

### Limitations

The study has limitations that may affect the interpretation of our results. First, inclusion criteria were participants who owned and used a smartphone and could speak and write Danish well enough to send text messages. Second, we explored the perspectives of 2 selected groups of patients undergoing orthopedic surgery. Therefore, the external validity of the results is unknown for other groups of patients undergoing orthopedic surgery, than the ones we explored.

In planning the study, we decided that initial interviews with patients and parents in phase 1, who were subsequently recruited to use eDialogue after discharge, were appropriate to identify patients’ perspectives on current communication pathways. However, some patients found it difficult to express themselves about this, as they had no or little previous experience of an orthopedic surgery context. In addition, there was a risk that the use of initial interviews combined with follow-up interviews within a short timespan (2 months) may have influenced the patients’ expressed attitudes in favor of the intervention in the follow-up interviews. Reflecting on this, it might have been better to perform initial interviews with a group of patients who were not given access to eDialogue afterwards.

In this study, we did not use log files to summarize the use data, as other studies have done [16,26], and this may be perceived as a limitation. However, we argue that log files, which report the number of log-in attempts, database entries, messages sent in total and the like, would not show the actual use as it presented to the participants in clinical practice. Therefore, manual counts were used to remove messages saying “thank you” or similar, as these are not considered relevant to the use of eDialogue in a health care setting.

Overall, the 24-hour weekday response time was met in this study and some patients reported extremely fast responses from HCPs. This finding must be interpreted with caution, as we cannot rule out that it is due to the Hawthorne effect, which suggests that people behave better when they are observed [46]. Conversely, it can also be an expression of the flexibility that lies in the digital asynchronous form of communication, giving HCPs the possibility to answer when they have the time for it, or it may simply reflect that the HCPs replied instantly (when able to) not to forget it. Nevertheless, an exclusively positive interpretation of compliance with the response time in this study may result in blindness toward the possible pitfalls that can occur in the real world if eDialogue is implemented. Insights from the perspective of HCPs can reveal this.

### Conclusions

The findings from this study indicate that the patients and the parents experienced an unmet need related to communication and collaboration following hospital discharge. eDialogue was overall evaluated positively, and the patients and parents perceived team-based digital communication as correspondent to their needs and suggested that it provided a sense of security after returning home. COC may be enhanced by assembling the team of HCPs in a simple digital communication solution with patients. However, eDialogue should be further evaluated and tested. Future research has to explore HCPs’ perspectives on the solution as well as establish the effects and organizational and economic incentives to use team-based digital communication in the context of orthopedic surgery care pathways.

## Appendix

Multimedia Appendix 1 Interview guides.

Multimedia Appendix 2 The number of patients from the deformity correction (DC) and anterior cruciate ligament (ACL) group who sent messages within the respective categories. HCP: health care professional.

## Abbreviations

ACL: anterior cruciate ligament
COC: continuity of care
DC: deformity correction
HCP: health care professional
