# Supplementary material for: Patient Perspectives on Communication Pathways After Orthopedic Surgery and Discharge and Evaluation of Team-Based Digital Communication: Qualitative Exploratory Study
Source: JMIR Hum Factors. 2024 Mar 29;11:e49696. doi: 10.2196/49696 (PMC11015373; doi:10.2196/49696)
Supplement: Multimedia Appendix 1 [file humanfactors_v11i1e49696_app1.docx]

# Supplementary material

**Interview guides**

In developing the interview guides for this study, we were inspired by the concept of continuity of care and the three types of continuity: informational, management and relational continuity. The table below provides an overview of the interview guides for phases 1 and 2 and highlights how the questions were related to continuity of care. Moreover, we used exploratory questions directly aimed at gaining insight into participants' experiences of using eDialogue for post-discharge communication. Throughout the interviews, probing questions were used to gain in-depth insight into the participants' perspectives and experiences, for example questions such as *"please give examples?",* *"can you elaborate?"* and *"if any, what was the problem in your opinion?".*

| **Phase 1 interviews** | **Phase 2 interviews** |
| --- | --- |
| **Current communication with HCPs** (questions inspired by informational continuity of care)  *"How do you experience communication with HCPs today surrounding your operation and discharge?*”  *"How do you experience the provision of information about your operation and discharge?* | **Experiences of using eDialogue for post-discharge communication** (questions inspired by informational continuity of care)  *"How have you experienced using eDialogue?"*  *“What significance (if any) has eDialogue had for you?”* |
| **Perspectives of current cross-disciplinary and cross-sectoral communication between HCPs (and you)** (questions inspired by management continuity of care)  *“How would you characterize the interdisciplinary and cross-sector collaboration and communication around your operation and discharge?”* | **Experiences related to interdisciplinary and cross-sector communication in eDialogue** (questions inspired by management continuity of care)  *"What significance (if any) has eDialogue had for the interdisciplinary and/or cross-sectoral communication between HCPs and you after discharge?"* |
| **Relations to healthcare professionals** (questions inspired by relational continuity of care)  *”How do you experience the relationships with the healthcare professionals involved in your treatment and care?”*  *"What does the relationship to healthcare professionals mean to you?"* | **Relations to healthcare professionals** (questions inspired by relational continuity of care)  *“How have you experienced the importance (if any) of being able to contact well-known healthcare professionals in eDialogue?”*  *”In what way (if any) did access to eDialogue have an impact on your relationships with the healthcare professionals?”* |
| **Expectations for using eDialogue** versus existing communication paths (exploratory questions related to the intervention)  *“What advantages and disadvantages do you expect from using eDialogue?”* | **Experiences of using or reasons for not using eDialogue** (exploratory questions related to the intervention)  *“Have you used eDialogue after discharge, and can you tell me about it (why/why not etc.)?”*  *“Have you called healthcare professionals by phone after discharge?” and “What made you choose traditional phone call over messaging in eDialogue?”*  **Perspectives for future use and suggestions for improvements**  *“Based on your experiences, what are your suggestions improvements and for future use of eDialogue?”* |
