# Supplementary figures and images for: Patient Perspectives on Communication Pathways After Orthopedic Surgery and Discharge and Evaluation of Team-Based Digital Communication: Qualitative Exploratory Study
Source: JMIR Hum Factors. 2024 Mar 29;11:e49696. doi: 10.2196/49696 (PMC11015373; doi:10.2196/49696)

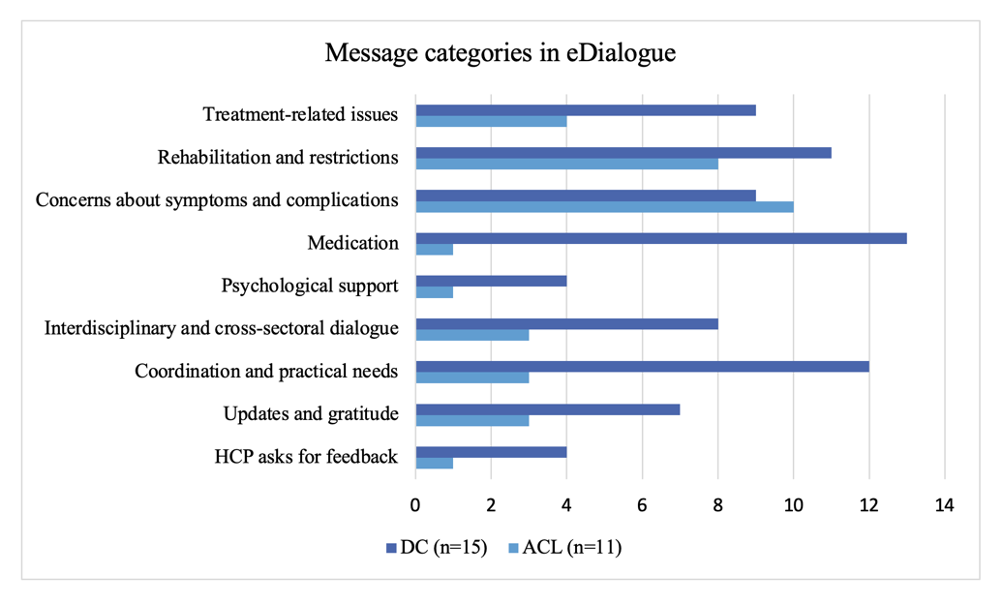

Supplement: Multimedia Appendix 2 [file humanfactors_v11i1e49696_app2.png]
